# Focus on Exercise Physiology and Sports Performance

**DOI:** 10.3390/life15010084

**Published:** 2025-01-12

**Authors:** Laikang Yu

**Affiliations:** 1 Beijing Key Laboratory of Sports Performance and Skill Assessment, Beijing Sport University, Beijing 100084, China; yulaikang@126.com; 2 Department of Strength and Conditioning Assessment and Monitoring, Beijing Sport University, Beijing 100084, China

## 1. Introduction

Exercise physiology is a crucial scientific discipline that explores the complex manner in which physical activity influences the physiological responses and adaptations of the human body [1,2]. With the development of exercise science, more emphasis has been placed on understanding the underlying physiological processes that can augment sports performance [3]. This is essential because performance hinges not only on technical skill and psychological factors but also on an individual’s physiological condition. Recent studies have demonstrated the complex interaction between various physiological systems during exercise, especially the cardiovascular, pulmonary, and metabolic responses, which are crucial for optimizing both performance and health outcomes [4,5,6].

Aerobic exercise, for instance, primarily demands energy through oxidative adenosine triphosphate (ATP) synthesis. The delicate balance between oxygen consumption and carbon dioxide production within muscle cells is closely related to maximal oxygen uptake (VO_2_max), a key indicator of aerobic capacity and a predictor of sports performance across diverse populations [7]. This correlation highlights the critical role of the cardiovascular and pulmonary systems in sustaining physical activity. Moreover, the physiological changes that occur with regular exercise, such as enhanced mitochondrial function and improved oxygen delivery, are pivotal in enhancing performance and overall health [8].

Contemporary research has further emphasized the importance of understanding the physiological reactions to various exercise modalities, including high-intensity interval training (HIIT) [9,10]. These forms of exercise elicit distinct metabolic responses and adaptations, influencing factors such as muscle hypertrophy, strength development, and metabolic flexibility [11,12]. Additionally, the significance of exercise in managing chronic diseases and neurodegenerative diseases has gained increasing recognition, making it a fundamental part of preventive healthcare [13,14].

In short, exercise physiology forms the basis for understanding how physical activity affects human health and performance. By exploring the physiological mechanisms involved, researchers and practitioners can develop more effective training programs and interventions to enhance sports performance and promote overall wellbeing.

## 2. An Overview of the Published Articles

The goal of the Special Issue, “Focus on Exercise Physiology and Sports Performance”, is to bring together the latest research that examines how the body adapts to exercise and provides insights into how training can enhance performance and optimize an athlete’s capabilities. Thirty-two manuscripts were submitted for consideration for the Special Issue, and thirteen papers were finally accepted for publication and inclusion. The key contents and findings of each paper are as follows.

Zhu et al. (contribution 1) explored how birth season and gender influence the gross and fine motor skills development of 2-year-olds. Their results indicated no gender effect on overall motor skills but a notable influence of birth season on fine motor quotient and total motor quotient. Notably, girls born during winter months demonstrated superior fine motor skills in comparison to those born in summer, hinting at crucial seasonal environmental impacts, especially for girls. These factors should be taken into account when designing early childhood programs aimed at advancing sports performance.

In a meta-analysis by Hu et al. (contribution 2), the effectiveness of isoinertial flywheel training (FWT) versus traditional resistance training (TRT) in augmenting muscle strength and power among healthy participants was examined. Their results confirmed that FWT is superior to TRT in enhancing muscle power, particularly when performed with squat and lunge exercises and within a specific session range, in both healthy untrained and well-trained individuals. Consequently, they advised coaches to integrate FWT into training programs for varied stimuli and improved power, suggesting a 6-week implementation with 2–3 sessions weekly, allowing at least a 48 h gap between sessions.

The study by Wang et al. (contribution 3) evaluated the immediate impact of various breath-hold (BH) scenarios on the aerobic fitness of 18 male elite rugby athletes. They found that among the various BH conditions, dynamic dry BH warm-up significantly improved aerobic fitness indicators such as peak oxygen uptake (VO_2_peak) and peak stroke volume (SVpeak) in elite rugby players, with these improvements strongly correlated with changes in red blood cells and hematocrit, suggesting that dynamic dry BH warm-up optimizes subsequent aerobic performance.

Ye et al. (contribution 4) designed and validated a comprehensive assessment framework for physical fitness in elite male badminton singles players, employing the Delphi method and analytic hierarchy process. This framework, comprising three primary, nine secondary, and twenty-one tertiary indicators, proved highly feasible and valid in assessing athletes’ fitness, providing practical guidance for enhancing competitive performance. This aids coaches in formulating targeted training plans and optimizing outcomes.

The study by Ieong et al. (contribution 5) investigated the relationship between musculoarticular stiffness and pedaling rate in sprint cycling, finding that participants with higher musculoarticular stiffness exhibited higher pedaling rates, peak crank force, and rate of crank force development, leading to higher power output in a 6 s sprint cycling test. The results suggest that optimizing cycling resistance or gear ratio to enhance these factors may be crucial for improving sprint cycling performance.

A meta-analysis conducted by Zhou et al. (contribution 6) focused on the impact of exercise on cancer-related fatigue in people with breast cancer, aiming to establish an optimal exercise prescription. They found that exercise interventions, particularly combining aerobic and resistance exercise conducted ≥ 3 times weekly for more than 60 min each session, totaling 180 min weekly, significantly improve cancer-related fatigue in breast cancer patients, with middle-aged patients benefiting the most. The optimal exercise prescription for breast cancer patients should incorporate combined exercise as the principal intervention.

In Hu et al.’s study (contribution 7), the effectiveness of inertial flywheel training (FWT) versus accentuated eccentric loading training (AELT) in augmenting neuromuscular performance among well-trained male college sprinters was examined. Their results showed that both training methods significantly improved lower-body strength, power, and speed. Notably, FWT demonstrated a superior ability to enhance the elastic energy storage and stretch-shortening cycle (SSC), as evidenced by greater improvements in countermovement jump (CMJ) and eccentric utilization ratio (EUR) compared to AELT.

The study by Zhou et al. (contribution 8) explored how transcranial direct current stimulation (tDCS) combined with resistance training influenced jump performance and brain activity using electroencephalography. Their results showed that the combination significantly enhanced vertical jump height and altered α-wave and β-wave power in frontal and temporal lobes, compared to either intervention alone.

The study by Ni et al. (contribution 9) analyzed changes in the electromyogram properties of agonist and antagonist muscles during a fatiguing heel-raise task. They found that during the initial stage of fatigue, both muscles exhibited increased activity and decreased mean frequency, with later differentiation in control strategies, where agonist muscle parameters stabilized, while antagonist muscle activity decreased. The findings suggest a differentiated control strategy by the central nervous system, enhancing the understanding of neuromuscular adaptations during fatigue and potential strategies for fatigue assessment and management.

Li et al. (contribution 10) investigated how stroboscopic visual conditions affected the key performance aspects of elite curling athletes. Their results showed that stroboscopic conditions significantly increased errors in these areas, suggesting that such conditions could be used as a training tool to enhance elite curling performance by challenging athletes to adapt and improve under limited visual information. Therefore, stroboscopic training has the potential to improve elite curling performance by enhancing the visual processing speed, reaction time, and motor skill control.

Bagchi et al. (contribution 11) found that the K-Deltas force platform exhibited high reliability and strong correlations with the other two tools, although small but consistent measurement differences were observed. Despite these discrepancies, the study highlights the K-Deltas force platform as a feasible choice for assessing CMJ, suggesting its potential use in monitoring training, predicting injury risk, assessing neuromuscular fatigue, and informing decision making in practical settings.

The study by Zhang et al. (contribution 12) examined the correlations between sprint force–velocity (Fv) characteristics and change of direction (COD), along with their impact on asymmetries in COD speed performance in volleyball and basketball players. Their results showed that the velocity (V_0_) and maximal ratio of force (RFmax) were crucial for improving COD performance, including linear sprints. Meanwhile, the force application technique (D_RF_), the force (F_0_), the ratio between F_0_ and V_0_ (Fv_slope_), and the maximal power (Pmax) collectively influenced the 180° COD performance. Additionally, the DRF and Fv_slope_ were important factors for asymmetries in COD speed performance.

Han et al. (contribution 13) conducted a meta-analysis to evaluate the impact of inspiratory muscle training (IMT) on chronic obstructive pulmonary disease (COPD) patients. By reviewing data from sixteen studies, they found that IMT significantly improved all three outcomes, and subgroup analysis revealed that IMT at < 60% maximal inspiratory muscle pressure (PImax), conducted for ≤ 20 min and more than 3 times per week, had the greatest benefits. Therefore, they recommended that COPD patients engage in IMT with these specific parameters to enhance their inspiratory muscle strength, reduce dyspnea, and improve their quality of life.

## 3. Conclusions

Exercise physiology involves many complex factors that greatly affects sports performance. Current research indicates that understanding key aspects such as energy systems, oxygen uptake, cardiovascular adaptations, muscle physiology, and effective recovery strategies is crucial for developing scientifically based training programs. These factors do not work independently; instead, they interact dynamically to shape an athlete’s overall abilities. Given these findings, a one-size-fits-all training method is not enough. Individual physiological responses to exercise can vary widely, highlighting the need for customized training regimens that account for an athlete’s unique characteristics. This requires ongoing research that not only explores the individual parts of exercise physiology but also introduces ideas from other fields like nutrition, psychology, and biomechanics.

Future studies should focus on personalized training methods, using new technology and data analysis to better understand how to best adapt training for different athletes. Additionally, using wearable devices and real-time monitoring tools can help with more precise recovery and training strategies, enhancing performance outcomes. As we move forward, it is crucial to consider all the different ideas and findings in the field. While some studies might focus more on certain physiological factors, having a complete view that recognizes how these systems all connect provides a more comprehensive understanding of sports performance. By working together across different fields and encouraging creative research, we can make large strides that not only benefit elite athletes but also improve health and fitness among the general population.
